# Endoscopic Closure After Colorectal ESD: A Literature Review and Meta-Analysis on Its Efficacy in Preventing Adverse Events

**DOI:** 10.3390/diagnostics16142148

**Published:** 2026-07-08

**Authors:** Naohisa Yoshida, Ken Inoue, Reo Kobayashi, Kazuya Maruo, Taku Kano, Katsuma Yamauchi, Hiroaki Kitae, Mayuko Seya, Mariko Kajiwara, Takeshi Yasuda, Naoto Iwai, Osamu Dohi, Kazuhiko Uchiyama, Yuri Tomita, Hardesh Dhillon, Rafiz Abdul Rani, Elsayed Ghoneem, Tomohisa Takagi

**Affiliations:** 1 Department of Molecular Gastroenterology and Hepatology, Graduate School of Medical Science, Kyoto Prefectural University of Medicine, 465 Kajii-cho, Kawaramachi-Hirokoji, Kamigyo-ku, Kyoto 602-8566, Japan; keninoue71@koto.kpu-m.ac.jp (K.I.); reipanna@koto.kpu-m.ac.jp (R.K.); takukano@koto.kpu-m.ac.jp (T.K.); hkitae@koto.kpu-m.ac.jp (H.K.); mayu0927@koto.kpu-m.ac.jp (M.S.); t-yasuda@koto.kpu-m.ac.jp (T.Y.); k-uchi@koto.kpu-m.ac.jp (K.U.);; 2 Department of Gastroenterology, Kyoto Hakuaikai Hospital, Kyoto 603-8465, Japan; yuri0428@koto.kpu-m.ac.jp; 3 Department of Gastroenterology, Barwon Health, Victoria 3220, Australia; hardeshdhillon92@gmail.com; 4 Gastroenterology Unit, Faculty of Medicine, Universiti Teknologi MARA, Bandar Puncak Alam 42300, Malaysia; rafiz@uitm.edu.my; 5 Hepatology and Gastroenterology Unit, Internal Medicine Department, Faculty of Medicine, Mansoura University, Mansoura 35516, Egypt; e_ghoneem@mans.edu.eg

**Keywords:** endoscopic submucosal dissection, colorectum, endoscopic closure, delayed bleeding, delayed perforation, post-endoscopic submucosal dissection coagulation syndrome

## Abstract

Endoscopic closure of mucosal defects after colorectal endoscopic submucosal dissection (ESD) has been increasingly adopted to prevent adverse events. Various devices and techniques, including conventional clips, clips with various technical tips, clip with line, clip with device, special clips, and suturing devices, have been developed, achieving high rates of complete closure (approximately 95% on average), although procedure times and technical complexity vary considerably. Among 14 eligible comparative studies, including randomized controlled trials and retrospective studies published up to April 2026, a meta-analysis was performed to evaluate the efficacy of complete closure after colorectal ESD in preventing delayed bleeding (DB), delayed perforation (DP), and post-ESD coagulation syndrome (PECS). For DB, pooled analysis demonstrated that complete endoscopic closure was significantly associated with a reduced risk of DB (odds ratio [OR]: 0.77, 95% confidence interval [CI]: 0.60–0.97, *p* = 0.030, I^2^ = 53.6%). In contrast, no significant differences were observed between the closure and non-closure groups for DP (OR: 0.60, 95% CI: 0.23–1.55, *p* = 0.290, I^2^ = 0.0%) or PECS (OR: 0.94, 95% CI: 0.65–1.38, *p* = 0.765, I^2^ = 56.6%). In a literature review, reported risk factors for DB include lesion size >50 mm, an ASA score of III or IV, antithrombotic therapy, age ≥75 years, and rectal location. Severe fibrosis and prolonged ESD procedure time have been reported as risk factors for DP, whereas female sex, age ≥70 years, right-sided colon, and lesion size >24 mm have been associated with PECS. Overall, various closure devices and techniques achieve high technical success rates after colorectal ESD. Complete closure appears beneficial for reducing DB, particularly in high-risk patients, whereas its efficacy for preventing DP and PECS remains uncertain. Further studies are warranted.

## 1. Introduction

Endoscopic submucosal dissection (ESD) enables a high rate of en bloc resection, thereby reducing local recurrence and allowing precise pathological assessment [1,2,3,4]. Although ESD is well established as a standard treatment for colorectal neoplasms, complications such as perioperative bleeding (PB), delayed bleeding (DB), perioperative perforation (PP), delayed perforation (DP), and post-ESD coagulation syndrome (PECS) remain important clinical concerns [2,3,4]. To mitigate the risk of these adverse events, various endoscopic closure after colorectal ESD has been increasingly adopted, with the choice of technique depending on patient risk factors and operator preference [5,6,7,8].

## 2. Adverse Events After ESD

The rates of PB, DB, DP, and PECS vary widely across studies, largely due to differences in definitions, ESD devices and strategies, geographic regions (Asia vs. Western countries), endoscopist’s skill, lesion location and size, and patient-related factors including anticoagulant use [2,3,4,5,6,7,8]. Definitions of adverse events actually differ among studies. In a representative Japanese study, PB was defined as severe bleeding requiring hemostatic forceps, whereas DB was defined as bleeding requiring endoscopic hemostasis or a ≥2 g/dL decrease in hemoglobin within 30 days after ESD [9]. While some studies have adopted this definition, others have defined DB solely as bleeding requiring endoscopic hemostasis [10]. PP has been defined as a complete defect in the muscular layer during the ESD procedure; however, its diagnosis is partly dependent on the operator’s subjective assessment. In contrast, DP is defined as no obvious perforation during the ESD procedure and no symptoms or free air on X-ray images immediately after ESD, but a subsequent sudden onset of abdominal pain and symptoms of peritoneal irritation accompanied by free air visible on X-ray or computed tomography [7,11]. PECS has been defined as localized abdominal pain (spontaneous pain and tenderness) accompanied by fever elevation (≥37.6 to 38.0 °C) or an inflammatory response (leukocytosis [≥10,000 or >12,000 cells/μL] or elevated C-reactive protein [≥0.5 mg or >3.0/dL]) without radiologic evidence of perforation, occurring more than 6 h after ESD [4,12].

In a large-scale systematic review including 104 studies and 13,833 colorectal ESD procedures, the rates of PB and DB were reported to be 0.39% (95% confidence interval (CI): 0.11–1.3%) and 1.8% (95% CI: 1.4–2.4%) in Asian countries, and 3.3% (95% CI: 1.4–7.6%) and 3.9% (95% CI: 2.5–5.8%) in European and North American countries, respectively [13]. In the same review, those of PP and DP were 3.8% (95% CI: 3.1–4.6%) and 0.18% (95% CI: 0.08–0.42%) in Asia, and 6.6% (95% CI: 4.6–9.4%) and 1.2% (95% CI: 0.29–4.6%) in Western countries, respectively [13]. A recent meta-analysis of 33 studies including 3958 colorectal ESD procedures in Western countries showed that the rates of DB and PP were 3.4% and 5.5%, respectively [14]. Regarding PECS, a systematic review including 19 studies (three prospective, two randomized controlled trials (RCT), and 14 retrospective studies) with 8151 patients who underwent colorectal ESD reported a pooled incidence of 8.9% [15]. Additionally, regarding differences in ESD techniques, a systematic review of eight RCTs comparing underwater ESD with conventional ESD reported DB rates of 3.1% and 2.5% (odds ratio [OR]: 1.34, 95% CI: 0.65–2.74, *p* = 0.43), PP rates of 4.1% and 4.6% (OR: 1.13, 95% CI: 0.64–2.00, *p* = 0.68), and PECS rates of 4.4% and 10.4% (OR: 0.38, 95% CI: 0.10–1.43, *p* = 0.15), respectively [16].

## 3. Various Closure Devices and Methods

**Figure 1:**
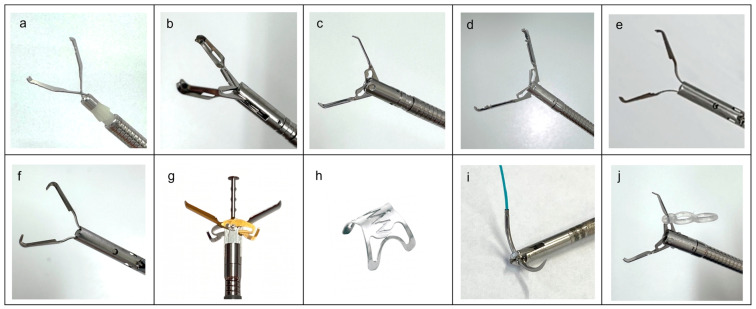
Various devices for closure of colorectal ESD defects. (**a**). EZ Clip, 8 mm (Olympus, Tokyo, Japan); (**b**). SureClip, 16 mm (Micro-Tech, Nanjing, China); (**c**). SureClip Eco, 11 mm (Micro-Tech, Nanjing, China); (**d**). Lockado, 20 mm (Micro-Tech, Nanjing, China); (**e**). Resolution 360, 11 mm (Boston Scientific, Marlborough, MA, USA); (**f**). Mantis Closure Device, 11 mm (Boston Scientific, Marlborough, MA, USA); (**g**). Dual Action Tissue Closure Device, 15 mm (Micro-Tech, Nanjing, China); (**h**). Over-the-Scope Clip (OTSC), 14 mm (Ovesco Endoscopy AG, Tübingen, Germany); (**i**). SutuArt (Olympus, Tokyo, Japan); and (**j**). SureClip Traction Band, 11 mm (Micro-Tech, Nanjing, China).

Various closure devices have been developed to prevent complications after colorectal ESD (Table 1). Endoscopic clips include both reopenable and non-reopenable types. In Japan, two non-reopenable clips, EZ Clip (Olympus, Tokyo, Japan) and Zeo Clip (Zeon Medical, Tokyo, Japan), can be reloaded onto a reusable deployment device, making them cost-effective (Figure 1a). Among regular-shaped reopenable clips, there are various sizes, shapes, and costs. SureClip (Micro-Tech, Nanjing, China) has a hole in its arm (8 mm clip and 16 mm clip), which facilitates use in clip-and-line techniques [6]. In contrast, the same company offers a lower-cost clip with a rotation function but without a hole in the arm, SureClip Eco (Micro-Tech, Nanjing, China) (Figure 1c). Its cost is approximately one-third that of the original SureClip. The largest opening width among reopenable clips is 20 mm, achieved by LOCKADO (Micro-Tech, Nanjing, China), and it has 10 teeth for secure closure (Figure 1d). Some clips, including SureClip (Micro-Tech, Nanjing, China), may fail to deploy properly if excessive pressure is applied to the defect base. Therefore, they should be applied gently under low insufflation. In contrast, clips such as Resolution and Resolution 360 (Boston Scientific, Marlborough, MA, USA) and StellaClip (Pentax, Tokyo, Japan) can be deployed regardless of the degree of pressure applied to the defect base (Figure 1e). Among special-shaped clips, Mantis Closure Device (MCD: Boston Scientific, Marlborough, MA, USA) has more sharply angled claws than regular clips, which help prevent slippage (Figure 1f). This clip is designed to grasp the mucosa firmly and enable closure of large defects. Another unique clip with two independently controlled arms is the Dual Action Tissue Closure Device (DAT, Micro-Tech, Nanjing, China) (Figure 1g). It is designed to facilitate closure of large defects, including full-thickness defects after endoscopic full-thickness resection (EFTR). The over-the-scope clip (OTSC: Ovesco Endoscopy AG, Tübingen, Germany) is used for closing deep defects such as muscle injury and perforation and can also be applied after EFTR (Figure 1h) [17]. However, it requires reinsertion after a dedicated device is mounted on the tip of the endoscope. Regarding suturing devices such as OverStitch (Boston Scientific, Marlborough, MA, USA), these systems use sutures and have a more complex structure than clips. Consequently, the procedure time is generally longer than that of clip-based methods. However, they provide durable closure and are typically used for muscle injury and EFTR. Other devices originally designed for different purposes have also been applied to defect closure, such as SureClip Traction Band (SCTB: Micro-Tech, Nanjing, China) as a traction device and Pneumo-Activate EVL Device as a ligation device for esophageal and gastric varices (Figure 1j) [18,19].

## 4. Efficacy of Various Closure Methods Using Various Devices

A literature search of PubMed up to April 2026 was performed using the key terms (“closure” “suture” OR “Clip”) AND (“endoscopic submucosal dissection” OR “ESD”) AND (“Colorectum” “Colon” OR “Colorectal”) to examine the efficacy of various closure methods in this review. Both single-arm studies and comparative studies on the efficacy of endoscopic prophylactic closure involving ≥5 cases were extracted. When a technique initially reported in fewer than 10 cases was later published in a study including more than 10 cases, the later report was included. The former one remained as appropriate. However, studies whose techniques and devices overlapped were excluded. Studies in which we could not extract the data of the colorectum from the overall data of several organs were also excluded. The clinical outcomes were measured as the rates of DB, DP, and PECS. Defect size, lesion size (when defect size was not available), complete closure rate, and closure time were also examined. According to these criteria, we reviewed 22 clinical studies evaluating various closure methods for colorectal ESD defects (Table 2) [20,21,22,23,24,25,26,27,28,29,30,31,32,33,34,35,36,37,38,39,40,41]. We classified reports about closure techniques into five types as follows: 1. Clip+TIPS, 2. Clip+Line, 3. Clip+Device, 4. Special Clip, and 5. Suturing device. Clip+TIPS is the type that does not need any special devices but needs some tips, such as incision, underwater situations, and muscle layer clipping. A representative report is the modified double-layer method (Origami method) [34]. Clip+Line is a clipping method with line, and a representative report is the reopenable clip-and-line method (ROLM) [37]. Clip+Device is a clipping method with a marketed accessory for other uses, and a representative report is clipping with SCTB (Micro-Tech, Nanjing, China) [41]. A special clip and suturing device are marketed for closure. Representatives are MCD and OverStitch, respectively [21,38]. Among 22 reports, the complete closure rate ranged from 73.0% to 100.0%, with an overall mean of 94.9%. Closure time ranged from 6.9 to 56.0 min, with a mean of 15.6 min. Most reports showed no incidence of DB or DP. Nine studies reported the incidence of PECS, which ranged from 0.0% to 9.8% (mean 3.1%). Although simple clip-only methods are convenient, they are often insufficient for complete closure of large defects. This limitation has led to the development of alternative techniques (Clips+TIPS), such as the mucosal incision method, hold-and-drag method, underwater clip closure, mucosa–submucosa clip closure, and the Origami method [20,22,26,27,34]. Clip-and-line techniques (Clip+Line), including ROLM, provide tight and reliable closure without creating dead space; however, they require additional time and technical expertise [25,30,31,36,37]. The use of various devices for closure, including both commercially available and handmade devices, was reported in the Clip+Device category. Among the special clips, the MCD achieved a median closure time of 6.9 min, which was the shortest among the 22 studies reviewed [38]. In the suturing devices category, these devices demonstrated moderate closure times (10.0–13.4 min), with the exception of SutuArt.

## 5. Case Presentations

**Figure 2:**
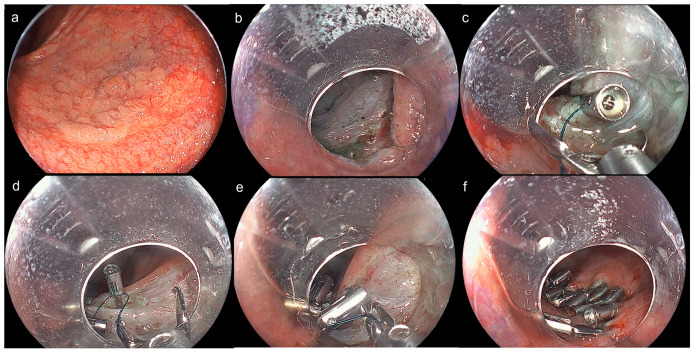
A case of complete closure using the Easy and Eco clip-over-the-line method (EOLM). (**a**) A 30 mm rectal lesion in the lower rectum (high-grade dysplasia); (**b**) It was resected en bloc using ESD; (**c**) In closure using SureClip Eco (Micro-Tech, Nanjing, China) with a line, a clip with line was first deployed on the distal side of the ulcer margin; (**d**) A second clip was inserted along the thread and placed at the proximal end of the mucosal defect; (**e**) The line was then pulled to approximate both edges with sequential clip deployment; (**f**) Finally the external end of the line was firmly tightened and cut using either an ESD knife or scissor-type forceps, achieving complete closure within 18 min.

Several representative methods are introduced. The Easy and Eco clip-over-the-line method (EOLM) is a modified version of the ROLM [42]. In ROLM, a specific reopenable clip with a hole in its arm, SureClip (8mm and 16 mm, Micro-Tech, Nanjing, China), is used in combination with a line. In contrast, EOLM allows the use of various types of clips. In a representative case, a 30 mm rectal lesion in the lower rectum (high-grade dysplasia) was resected, and SureClip Eco (Micro-Tech, Nanjing, China) with a line, which don’t have a holw in its arm, was used for closure (Figure 2a,b). A clip was first deployed on the distal side of the ulcer margin, and the next clip was inserted along the thread and placed at the proximal side of the mucosal defect (Figure 2c). The line was then pulled to approximate the proximal and distal edges of the defect, and additional clips were sequentially deployed (Figure 2d). By gradually tightening the line, the defect was progressively closed (Figure 2e). Finally, the external end of the line was firmly pulled to achieve complete closure, and the line was cut using either an ESD knife or scissor-type forceps (Figure 2f).

**Figure 3:**
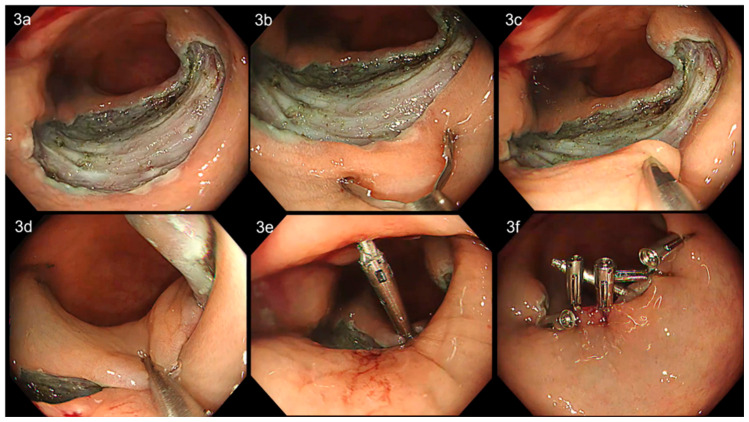
A case of complete closure using the Mantis Closure Device (MCD). (**a**) A 35 mm lesion in the sigmoid colon was resected using ESD; (**b**) The surrounding normal mucosa on the anal side of the defect was grasped using MCD; (**c**) The device was rotated 90 degrees; (**d**,**e**) The anal-side mucosa was pushed toward the oral-side mucosa, and the MCD was reopened and deployed while maintaining its grip to approximate the mucosal edges; (**f**) Additional deployment of SureClips (width: 16 mm, Micro-Tech, Nanjing, China) was performed to achieve complete closure with a total closure time of 6 min.

The MCD is a reopenable clip with sharp claws and a width of 11 mm (Figure 1f). In a representative case, a 35 mm lesion in the sigmoid colon was resected by ESD (Figure 3a). After ESD, the surrounding normal mucosa on the anal side of the defect was grasped using MCD (Figure 3b). The device was then rotated 90 degrees by both the operator and assistant (Figure 3c) [43]. Subsequently, the anal-side mucosa was pushed toward the oral-side mucosa, similar to previously described techniques using conventional clips. The MCD was reopened and deployed while maintaining its grip on the anal-side mucosa (Figure 3d), thereby approximating the mucosal edges (Figure 3e). Additional SureClips (width: 16 mm, Micro-Tech, Nanjing, China) were then deployed to achieve complete closure, with a total closure time of 6 min (Figure 3f).

**Figure 4:**
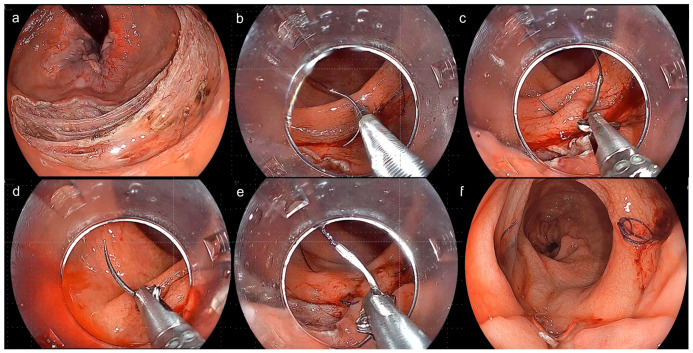
A case of complete closure using SutuArt. (**a**) A 40 mm T1b cancer in the lower rectum was treated with ESD, in which resection extended into the muscular layer to achieve histopathological complete resection. (**b**) SutuArt was delivered to the rectum with the needle secured within a large distal cap to conceal the needle tip, and after placement of an initial anchoring suture, a second suture was performed on the oral side of the defect. (**c**,**d**) The needle was released and subsequently re-grasped. (**e**) The mucosal edges were sequentially sutured in a zigzag fashion at 5–10 mm intervals under endoscopic visualization to approximate the defect. (**f**) Complete closure was achieved within 25 min, after which the remaining suture and needle were cut using scissor-type forceps.

SutuArt is a unique suturing device that uses a surgical needle and suture (Figure 1i). In a representative case, ESD was performed for a 40 mm T1b cancer in the lower rectum, with resection extending into the muscular layer to achieve histopathological complete resection (Figure 4a). A therapeutic single-channel colonoscope and a V-Loc 180 absorbable barbed suture (VLOCL0604; Covidien, Mansfield, MA, USA) were used. The tail of the suture was manually knotted. The suture was delivered to the rectum with the needle secured within a large distal cap to protect the needle tip. After an initial anchoring stitch was placed, a second stitch was placed on the oral side of the defect (Figure 4b). The needle was released from SutuArt and subsequently re-grasped (Figure 4c,d). The mucosal edges were then sutured in a zigzag fashion at 5–10 mm intervals under endoscopic visualization, enabling approximation of the defect edges (Figure 4e). Finally, complete closure was achieved, and the remaining suture and needle were cut using scissor-type forceps (Figure 4f).

**Figure 5:**
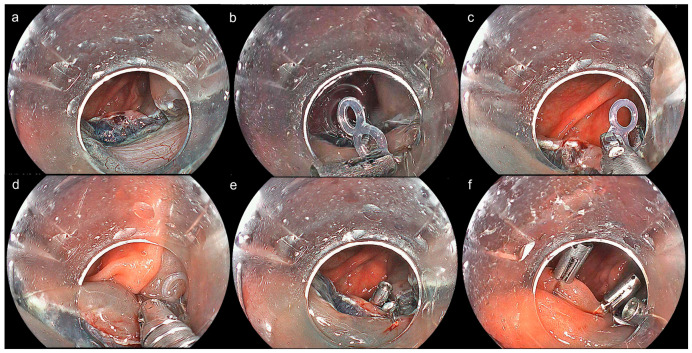
A case of complete closure using SureClip Traction Band (SCTB). (**a**) A 35 mm cecal lesion was resected using ESD; (**b**) SCTB was deployed on the normal mucosa at the anal side of the defect; (**c**) The silicone band was grasped using a reopenable clip (SureClip Eco, Micro-Tech, Nanjing, China), preferably at the tip of the second band; (**d**) The clip was advanced toward the oral side while maintaining traction; (**e**) The clip was deployed on the normal mucosa at the oral side of the defect; (**f**) Additional reopenable clips were deployed to achieve complete closure within 9 min.

The SCTB (Micro-Tech, Nanjing, China) is a traction device consisting of two interconnected elastic silicone bands pre-mounted on a standard 11 mm hemostatic clip (Figure 1i). In a representative case, ESD was performed for a 35 mm cecal lesion (Figure 5a). The SCTB was first deployed on the normal mucosa at the anal side of the defect (Figure 5b). The silicone band was then grasped using SureClip Eco (Micro-Tech, Nanjing, China), preferably at the tip of the second band (Figure 5c). The clip was advanced toward the oral side while maintaining traction (Figure 5d) and deployed on the normal mucosa at the oral side of the defect (Figure 5e). Additional reopenable clips were subsequently deployed to achieve complete closure (Figure 5f).

These representative techniques highlight the versatility of available closure strategies tailored to defect size, location, and procedural complexity.

## 6. Efficacy of Complete Closure for DB, DP, and PECS According to Reported Studies Including Randomized Controlled Trials and Retrospective Studies: A Meta-Analysis

### 6.1. Methods

We searched PubMed, Embase, and the Cochrane Library for eligible studies published up to April 2026 to perform a meta-analysis evaluating the efficacy of endoscopic closure after colorectal ESD. The search terms included (“closure” OR “suture” OR “clip”) AND (“endoscopic submucosal dissection” OR “ESD”) AND (“colorectum” OR “colon” OR “colorectal”). The efficacy of endoscopic closure in DB, DP, and PECS was evaluated.

The number of adverse events and total patients in the closure and non-closure groups was extracted from each eligible study. Definitions of complete closure and adverse events were accepted as reported in the original studies.

The inclusion criteria were as follows: (i) patients undergoing colorectal ESD; (ii) prophylactic endoscopic closure after ESD; (iii) the presence of a non-closure control group; (iv) reported incidence of DB, DP, or PECS; (v) RCTs or observational studies (prospective or retrospective cohort studies and case–control studies); and (vi) publication in English.

Two investigators (N.Y. and Y.T.) independently extracted the data and assessed study quality. Any discrepancies were resolved through discussion.

### 6.2. Statistical Analysis

Odds ratios (ORs) with 95% CIs were calculated for each outcome. When a study contained a zero-event cell, a continuity correction of 0.5 was applied. Studies with no events in either group were excluded from pooled effect estimation for the corresponding outcome.

Pooled analyses were performed using a random-effects model according to the DerSimonian–Laird method because clinical and methodological heterogeneity among studies was anticipated. Statistical heterogeneity was assessed using Cochran’s Q test and the I^2^ statistic, with an I^2^ value >50% considered indicative of substantial heterogeneity.

Forest plots were generated for DB, DP, and PECS, including study weights, pooled estimates, and 95% CIs. Because definitions of adverse events varied slightly among the included studies, the original definitions used in each study were accepted. Statistical analyses and forest plots were generated using Review Manager (RevMan) version 5.4 and Python version 3.10.

### 6.3. Results

A total of 520 studies were identified through the database search. After screening and eligibility assessment, 14 studies were included in the meta-analysis (Table 3) [25,41,44,45,46,47,48,49,50,51,52,53,54,55].

The meta-analysis evaluated the incidence of DB, DP, and PECS in patients undergoing colorectal ESD by comparing closure and non-closure groups. Among the 14 included studies, four were RCTs and 10 were retrospective observational studies. The methodological quality of the four RCTs was assessed using the Cochrane Risk of Bias 2 tool (Supplementary Table S1) [56]. Additionally, those of the 18 observational studies were assessed according to the Newcastle–Ottawa Scale (Supplementary Table S2) [57].

**Figure 6:**
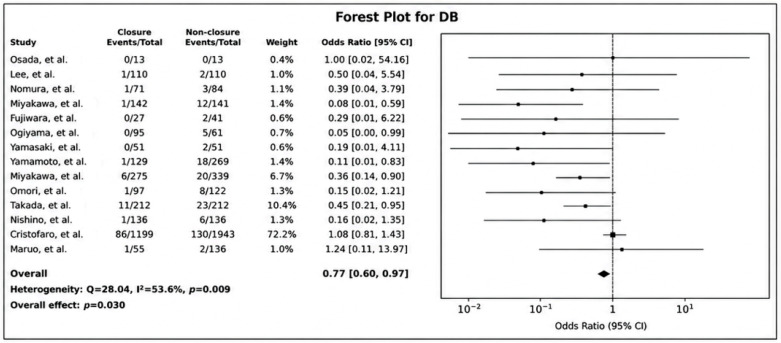
Forest plot of the included studies evaluating the efficacy of complete closure for DB after colorectal ESD. DB: delayed bleeding, ESD: endoscopic submucosal dissection [25,41,44,45,46,47,48,49,50,51,52,53,54,55].

**Figure 7:**
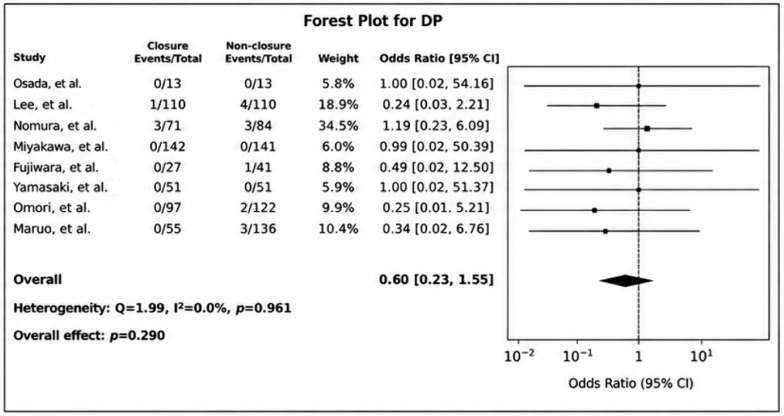
Forest plot of the included studies evaluating the efficacy of complete closure for DP after colorectal ESD. DP: delayed perforation, ESD: endoscopic submucosal dissection [25,41,44,45,46,47,48,52].

**Figure 8:**
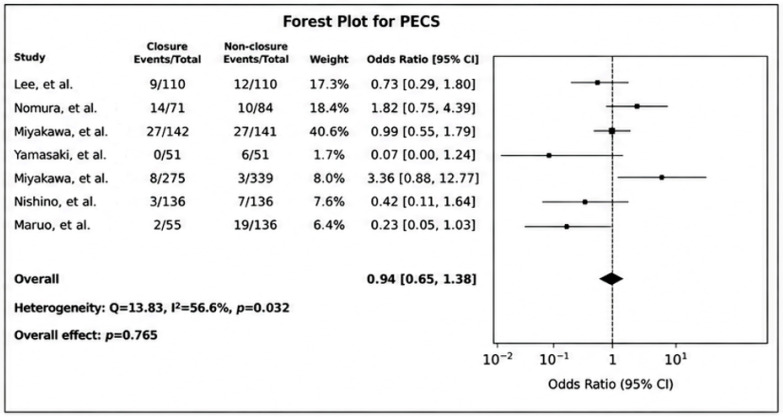
Forest plot of the included studies evaluating the efficacy of complete closure for PECS after colorectal ESD. PECS: post-ESD coagulation syndrome, ESD: endoscopic submucosal dissection [25,41,45,46,47,51,54].

Regarding the meta-analysis, for DB, pooled analysis demonstrated that endoscopic complete closure was significantly associated with a reduced risk of DB (OR: 0.77, 95% CI: 0.60–0.97, *p* = 0.030, I^2^ = 53.6%) (Figure 6). For DP, there was no significant difference between the closure and non-closure groups (OR: 0.60, 95% CI: 0.23–1.55, *p* = 0.290, I^2^ = 0.0%) (Figure 7). For PECS, endoscopic closure was not significantly associated with risk reduction (OR: 0.94, 95% CI: 0.65–1.38, *p* = 0.765, I^2^ = 56.6%) (Figure 8). 

Among these 14 studies, the overall rates of DB, DP, and PECS were 4.3% vs. 6.6% (*p* < 0.001), 0.6% vs. 1.6% (*p* = 0.109), and 7.5% vs. 8.5% (*p* = 0.421) in the closure and non-closure groups (Table 4). In the four RCTs, closure significantly reduced DB (0.9% vs. 4.9%, *p* = 0.004), whereas no significant differences were observed for DP (1.2% vs. 2.0%, *p* = 0.582) or PECS (15.5% vs. 15.2%, *p* = 0.913). In the 10 retrospective studies, closure was also associated with a lower rate of DB (4.7% vs. 6.8%, *p* < 0.001) and a reduced rate of PECS (2.5% vs. 5.3%, *p* = 0.024), while DP remained similar between groups (0.0% vs. 1.2%, *p* = 0.085).

Among the four RCTs, lesion size was generally comparable between groups. Osada et al. reported no DB or DP in either group (Table 3) [44]. Lee et al. demonstrated low rates of DB, with no significant difference between the closure and non-closure groups (0.9% vs. 1.8%, *p* = 1.000), and no significant differences were observed for DP (0.9% vs. 3.6%, *p* = 0.365) or PECS (8.2% vs. 10.9%, *p* = 0.491) [45]. Similarly, Nomura et al. reported no significant differences in DB (1.4% vs. 3.6%, *p* = 0.735), DP (4.2% vs. 3.6%, *p* = 0.835), or PECS (19.7% vs. 11.9%, *p* = 0.180) [46]. In contrast, Miyakawa et al. demonstrated a significantly lower rate of DB in the closure group compared with the non-closure group (0.7% vs. 8.5%, *p* = 0.001), while no differences were observed for DP (0% vs. 0%, *p* = 1.000) or PECS (19.0% vs. 19.1%, *p* = 1.000) [47]. Overall, earlier RCTs did not demonstrate a clear benefit of closure, whereas a more recent trial suggested a reduction in DB.

In the 10 retrospective studies, closure was more frequently associated with lower rates of DB (Table 3) [25,41,48,49,50,51,52,53,54,55]. Ogiyama et al. reported a significantly lower DB rate in the closure group compared with the non-closure group (0.0% vs. 8.9%, *p* = 0.012), and Yamamoto et al. also showed a significant reduction (0.8% vs. 6.7%, *p* = 0.019) [49,50]. Miyakawa et al. reported similar findings (2.2% vs. 5.9%, *p* = 0.022) [51]. Takada et al. demonstrated significantly lower DB in the closure group among patients receiving antithrombotic therapy, both in those receiving direct oral anticoagulants (5.2% vs. 10.8%, *p* = 0.048) and warfarin (6.1% vs. 17.1%, *p* = 0.049) [53]. In contrast, Cristofaro et al. reported no significant difference between groups (7.2% vs. 6.7%, *p* = 0.66) [55].

DP was rare across studies, with most reporting no events or no significant differences, such as Omori et al. (0.0% vs. 1.6%, *p* = 0.581) and Nishio et al. (0.0% vs. 0.0%, *p* = 1.000) [52,54]. Regarding PECS, the results were inconsistent. Yamasaki et al. demonstrated a significantly lower rate in the closure group (0.0% vs. 11.8%, *p* = 0.035), and Maruo et al. also reported a significant reduction (3.6% vs. 14.0%, *p* = 0.042) [25,41]. However, other studies did not show significant differences, such as Nishio et al. (2.2% vs. 5.1%, *p* = 0.335), and Miyakawa et al. reported a non-significant trend toward higher rates in the closure group (2.9% vs. 0.9%, *p* = 0.115) [51,54].

### 6.4. Limitations

The present work should be regarded as a meta-analysis and not a formal PRISMA-compliant meta-analysis. Although a structured literature search, study selection, quality assessment, and random-effects pooled analyses were performed, a PRISMA flow diagram and formal publication-bias assessment were not included. Therefore, the findings should be interpreted as those of a literature review with meta-analysis rather than a formal systematic review and meta-analysis. The definitions of DB, DP, and PECS were not completely uniform among the included studies, which may have contributed to heterogeneity. Most included studies were retrospective observational studies, and only a limited number of RCTs were available. Closure techniques and devices differed among studies, including conventional clips, clip-and-line methods, and other closure devices. Regarding DP, its incidence is considerably lower than that of DB and PECS; therefore, a larger number of cases may be required to clarify whether complete closure reduces its occurrence.

## 7. Risk Factors of DB, DP, and PECS

DB after colorectal ESD has been associated with several patient- and procedure-related factors. Ogasawara et al. identified continued antithrombotic therapy as an independent risk factor for DB (OR: 3.67, 95% CI: 1.22–11.02, *p* = 0.021) [58]. In addition, tumor size ≥30 mm (OR: 2.85, 95% CI: 1.34–6.05, *p* = 0.006) and rectal location (OR: 2.18, 95% CI: 1.01–4.71, *p* = 0.047) have been reported as significant predictors [59]. Longer procedure time has also been suggested to increase the risk of DB, although results are not entirely consistent across studies. A study proposed a risk scoring system for DB. The risk factors for DB after colorectal ESD were identified as follows: lesion size >50 mm (OR: 3.63, 95%CI: 2.02–7.14, three points), an ASA score of III or IV (OR: 2.26, 95% CI: 1.32–3.92, two points), use of antithrombotic agents (OR: 1.72, 95% CI: 1.01–2.94, one point), age ≥75 years (OR: 1.60, 95% CI: 0.95–2.70, one point), and rectal location (OR: 1.51, 95% CI: 0.92–2.48, one point) [60]. Based on the cumulative predictive score, the incidence of delayed bleeding was 4.1% in the low-to-medium risk group (0–4 points) and 17.5% in the high-risk group (5–8 points).

DP is really rare, and large-scale studies about its risk factors are limited. However, in our previous multicenter study of 4632 colorectal ESD procedures from 13 institutions, the incidence of DP was 0.39% [61]. Multivariate analysis identified severe fibrosis (OR: 4.61, 95% CI: 1.50–14.20, *p* = 0.007), longer ESD procedure time (OR: 1.01, 95% CI: 1.00–1.02, *p* = 0.042), and complete closure (OR: 0.12, 95% CI: 0.01–0.96, *p* = 0.046) as significant factors associated with DP. Accordingly, complete closure may reduce the risk of DP.

PECS is associated with transmural thermal injury. Arimoto et al. reported that right-sided colon location (OR: 3.23, 95% CI: 1.49–7.01, *p* = 0.003), tumor size ≥30 mm (OR: 2.18, 95% CI: 1.01–4.71, *p* = 0.047), and female sex (OR: 1.95, 95% CI: 1.02–3.73, *p* = 0.043) were independent risk factors [62]. Similarly, Ito et al. identified longer procedure time (OR: 1.02 per minute, 95% CI: 1.01–1.03, *p* < 0.001) as a significant predictor of PECS [63]. Collectively, these findings indicate that DB, DP, and PECS share common risk factors related to procedural complexity and thermal injury during ESD. In particular, factors such as larger lesion size, prolonged procedure time, and difficult dissection conditions (e.g., fibrosis) appear to play central roles across these adverse events. Our recent study made a five-variable PECS-predictive scoring system (FPSS) for early post-procedure prediction of PECS [64]. Multivariate analysis identified five independent predictors of PECS: right colon (OR: 3.55, *p* = 0.004), elderly female ≥70 years (OR: 2.52, *p* = 0.022), body temperature ≥37.6 °C (OR: 16.70, *p* < 0.001), WBC >8000/µL on day 1 (OR: 13.78, *p* < 0.001), and lesion size >24 mm (OR: 3.03, *p* = 0.003). The FPSS (range 0–9) achieved an AUC of 0.881, with high negative predictive value (97.4%). These observations underscore the importance of meticulous technique and targeted preventive strategies, including appropriate energy control and selective mucosal closure, especially in high-risk cases.

## 8. Indications for Endoscopic Closure Based on Reported Evidence and Clinical Factors

Previous reviews and our meta-analysis demonstrated that complete closure significantly reduces DB after colorectal ESD, providing strong evidence for its use [4,5,6,7,8]. However, the overall incidence of DB remains relatively low, occurring in 4.3% and 6.6% of patients in the closure and non-closure groups, respectively (Table 4). In contrast, certain high-risk factors substantially increase the risk of DB, including lesion size >50 mm (OR: 3.63), ASA class III–IV (OR: 2.26), and antithrombotic therapy (OR: 1.72) [60]. Our previous study also demonstrated the efficacy of complete closure in patients receiving anticoagulants, with DB rates of 5.2% and 10.8% in the closure and non-closure groups, respectively [53]. Therefore, complete closure should be strongly considered in high-risk cases, whereas its indication in low-risk cases may be determined individually.

The choice of closure method should be based on defect size, cost, procedure time, operator expertise, and local availability. Most closure techniques have been evaluated for defects <50 mm (Table 2), where procedural efficiency and cost-effectiveness are important considerations. Our previous study reported a median closure time of 6.9 min for defects measuring 20–57 mm, suggesting that this method may be a practical option for defects <50 mm [43]. In contrast, only a limited number of techniques are suitable for defects >50 mm, including suturing devices, the Origami method, and ROLM.

Durable closure may be particularly important in patients receiving antithrombotic agents because DB tends to occur later in these patients [43]. Recent closure techniques using dedicated devices have achieved prolonged maintenance of defect closure. For example, MCD and the SureClip Traction Band maintained complete closure in all cases two days after ESD [38,41], while ROLM and OTSC have also demonstrated durable closure [65,66].

Although complete closure did not significantly reduce DP, this may be attributable to the low incidence of DP and insufficient statistical power. In clinical practice, we preferentially perform complete closure in lesions at high risk of DP, particularly large right-sided lesions and those requiring extensive coagulation near the muscularis propria. However, both DP and PECS are partly caused by thermal injury to the muscle layer during dissection and hemostasis. Consistent with this mechanism, many studies have not demonstrated a significant reduction in PECS with complete closure (Table 3). Therefore, strategies aimed at minimizing thermal injury, such as lower electrosurgical settings and underwater techniques, may be more effective for preventing DP and PECS [67,68,69]. Further studies are expected to prove it. 

## 9. Conclusions

A wide range of closure devices and techniques is available, achieving high technical success rates in defect closure after colorectal ESD. Individual studies suggest that endoscopic closure is associated with a reduction in DB in many settings, although results from earlier RCTs were inconsistent. Pooled analyses confirm that closure significantly reduces DB in both randomized and retrospective data. Complete closure is recommended for preventing DB according to the patients’ risks. In contrast, the incidence of DP is low and does not appear to be significantly affected by closure. The effect on PECS is also controversial. Thus, further well-designed studies including RCTs are warranted to clarify the role of closure in preventing DP and PECS.

## Figures and Tables

**Table 1:** Various devices for closure after colorectal ESD.

Type of Clip	Brand Name	Company	Size (mm)	Required Channel Diameter	Reinsertion
No-reopenable clip (reloaded)	Ez clip	Olympus	6/8/11	2.8	
Partially reopenable clip (reloaded)	Zeo clip	Zeon Medical	11	2.8	
Reopenable clip	QuickClip2	Olympus	9	2.8	
Reopenable clip	Retentia	Olympus	9/12/16	2.8	
Reopenable clip	Sure Clip	Micro-Tech	8/11/16	2.8	
Reopenable clip	Sure Clip Eco	Micro-Tech	11	2.8	
Reopenable clip	LOCKADO	Micro-Tech	20	2.8	
Reopenable clip	Resolution	Boston Scientific	11	2.8	
Reopenable clip	Resolution360	Boston Scientific	11	2.8	
Reopenable clip	StellaClip	Pentax Medical	11/13	2.8	
Reopenable clip	Hemoclip	Anrei	9/11/13/16	2.8	
Reopenable clip	VedClip	Vedkang	11/13/16	2.8	
Reopenable clip	ClearEndoClip	FINEMEDIX	11/12/15/16	2.8	
Reopenable clip	MENFIS	MENFIS Lorea			
Reopenable clip	Assurance Clip	STERIS	9/11//13/16/18	2.8	
Reopenable clip with sharp claw	Mantis Closure Device	Boston Scientific	11	2.8	
Reopenable clip with sharp claw	Instinct Plus Clip	Cook Medical	16	2.8	
Special-shaped clip	Dual Action Tissue Closure Device	Micro-Tech	15	3.2	
Special-shaped clip	OTSC	Ovesco Endoscopy AG	Mini/11/12/14	2.8	Yes
Special-shaped clip	OTS Padlock Clip	Aponos Medical	Mini/11/14	2.8	Yes
Suturing	OverStitch	Boston Scientific		3.2	Yes
Suturing	SutuArt	Olympus		3.2	Yes
Suturing	X-tack Helix	Boston Scientific		2.8	
Reopenable clip with band (Traction device)	SureClip Traction Band	Micro-Tech	13	3.2	
Reopenable clip with band (Traction device)	SO clip	Zeon Medical	11	2.8	
Endoscopic O-ring (ligation device for varices)	Pneumo-Activate EVL Device	Sumitomo bakelite			
Endoloop (ligation device for polypectomy)	Endoloop	Olympus			

ESD: endoscopic submucosal dissection.

**Table 2:** Clinical reports for various devices for closure after colorectal ESD.

Name of Closure	Type	Author	Year	Number	Defect Size, Median [Range]	Complete Closure Rate	Closure Time, Median [Range]	DB Rate	DP Rate	PECS Rate
Regular clip with mucosal incision	Clip+TIPS	Otake et al. [20]	2012	10	26.8: [8.0–50.0] *	100.0	15.0 [8.0–35.0]	0	0	N/A
Endoscopic suturing (OverStitch, 1st report)	Suturing Device	Kantsevoy et al. [21]	2014	8	40.0 [30.0–80.0] *	100.0	10.0 ± 5.8 **	0	0	N/A
Hold and drag	Clip+TIPS	Akimoto et al. [22]	2016	19	40.2 ± 12.0 **	94.7	10.7 ± 7.2 **	0	0	N/A
Endoscopic Slip knot clip suturing	Clip+Line	Nishizawa et al. [23]	2017	7	35 [15.0–46.0]	100.0	17.7 [12.9—20.7]	0	0	0
Pre-detached endoloop strategy	Ciip+Device	Wang et al. [24]	2017	18	28.0 [12.0–50.0] *	100.0	13.5 [8.0–20.0]	0	0	0
Line-assisted closure	Clip+Line	Yamasaki et al. [25]	2018	61	35.0 [23.0–73.0]	95.0	14.0 [6.0–35.0]	0	0	2.0
Underwater	Clip+TIPS	Yamasaki, et al. [26]	2019	21	31.0 [18.0–47.0] *	100.0	11.0 [6.0–21.0]	0	0	N/A
Mucosa– submucosa clip closure	Clip+TIPS	Nishizawa et al. [27]	2018	25	31.2 ± 1.1 **	96.0	9.6 ± 4.4 **	0	0	N/A
Clip-on clip closure	Clip+TIPS	Nomura et al. [28]	2020	32	34.0 [28.0–73.0]	97.0	8.0 [3.5–29.2]	0	0	N/A
Double- layer method	Clip+TIPS	Abiko et al. [29]	2020	26	32.0 (20.0–24.0) ***	88.5	20.0 [16.0–34.0]	0	0	3.8
Endoscopic suturing (SutuArt)	Suturing Device	Abe et al. [30]	2020	11	38.0 [25.0–55.0]	73.0	56.0 [30.0–120.0]	9	0	N/A
Endoscopic suturing (OverStitch, 2nd report)	Suturing Device	Han et al. [31]	2020	13	38.7 ± 16.1 **	100.0	13.4 ± 5.9 **	0	0	N/A
Loop and Open-Close Clips	Clip+Device	Yoshida et al. [32]	2021	9	40.9 ± 8.2 **	100.0	18.7 ± 8.0 **	0	0	N/A
Continuous barbed suturing line technique	Clip+Line	Chu et al. [33]	2023	31 including EMR	34 (28.0–49.0) ***	93.5	13 (11.0–16.0) ***	0	0	3.2
Modified double-layer method (Origami method)	Clip+TIPS	Masunaga et al. [34]	2023	47	38 [25.0–85.0]	94	17 [9.0–37.0]	0	0	4
Endoscopic suturing (X-tack helix)	Suturing Device	Farha et al. [35]	2023	82	30.0 (25.0–40.0) ***	92.7	10.0 (6.3–17.3) ***	1.2	0.0	N/A
Endoscopic Ligation with O-Ring Closure	Clip+Device	Tada et al. [36]	2023	30 only rectal ESD	29.0	83.3	25.5 (20.0–30.0) ***	3.3	0	N/A
Reopenable clip-over-the-line method	Clip+line	Nomura et al. [37]	2023	30	45 [35.0–70.0]	100.0	25.0 [14.0–52.0]	0	0	N/A
MANTIS	Special Clip	Yoshida et al. [38]	2024	61	32.3 [20.0–57.0] *	98.4	6.9 [3.0–15.0]	0	0	9.8
Loop 9	Clip+Device	Tanabe et al. [39]	2024	118	30.0 [15.0–74.0] *	96.6	14.0 [11.25–17.0] ***	0	0	1.7
Dual action tissue closure	Special Clip	Mohammed et al. [40]	2024	107, including 63 EMR	40.0 (30.0–45.0) #	96.3	7.0 (5.0–10.0) ***	0.9	0	N/A
Traction device-assisted complete closure	Clip+Device	Maruo et al. [41]	2026	55	35.5 [20.0–60.0]	98.2	8.6 [4.9–32.8]	1.8	0	3.6

ESD: endoscopic submucosal dissection, EMR: endoscopic mucosal resection, DB: delayed bleeding, DP: delayed perforation, PECS: post-ESD coagulation syndrome, N/A: not applicable, * lesion size, median [range], ** mean ± SD, *** median (interquartile range: IQR), # lesion size median (IQR).

**Table 3:** Clinical reports the comparison between the closure group and non-closure for DB, DP, and PECS after colorectal ESD.

Authors, Year	Published Year	Country	Study Design	Resected Lesion Size (mm), Mean ± SD Closure/Non-closure	Closure Method	Case Number Closure/Control	DB Closure vs. Non-Closure, % (n)	DP Closure vs. Non-Closure, % (n)	PECS Closure vs. Non-Closure, % (n)
Osada et al. [44]	2016	Japan	RCT	677 ± 306/ 790 ± 221	Regular clip	13/13	0.0 (0) vs. 0.0 (0) *p* = 1.000	0.0 (0) vs. 0.0 (0) *p* = 1.000	N/A
Lee et al. [46]	2019	Korea	RCT	19.7 ± 8.5/ 22.9 ± 9.8 *	Regular clip	110/110	0.9 (1) vs. 1.8 (2) *p* = 1.000	0.9 (1) vs. 3.6 (4) *p* = 0.365	8.2 (9) vs. 10.9 (12) *p* = 0.491
Nomura et al. [45]	2020	Japan	RCT	22 (20–28)/ 20 (20–30) **	Regular clip	71/84	1.4 (1) vs. 3.6 (3) *p* = 0.735	4.2 (3) vs. 3.6 (3) *p* = 0.835	19.7 (14) vs. 11.9 (10) *p* = 0.180
Miyakawa et al. [47]	2025	Japan	RCT	38.9 ± 11.2/ 40.0 ± 10.7	Regular clip	142/141	0.7 (1) vs. 8.5 (12) *p* = 0.001	0.0 (0) vs. 0.0 (0) *p* = 1.000	19.0 (27) vs. 19.1 (27) *p* = 1.000
Fujiwara et al. [48]	2013	Japan	Ret	32 (16–55)/ 35 (18–70) #	Regular clip	27/41	0.0 (0) vs. 4.9 (2) *p* = 0.666	0.0 (0) vs. 2.4 (1) *p* = 0.832	N/A
Yamasaki et al. [25]	2018	Japan	Ret	35 (23–75)/ 30 (20–70) **	Line and clip	51/51	0.0 (0) vs. 3.9 (2) *p* = 0.475	0.0 (0) vs. 0.0 (0) *p* = 1.000	0.0 (0) vs. 11.8 (6) *p* = 0.035
Ogiyama et al. [49]	2018	Japan	Ret	23.5 ± 7.0/ 22.2 ± 9.0 *	Regular clip	95/61	0.0 (0) vs. 8.9 (5) *p* = 0.012	N/A	N/A
Yamamoto et al. [50]	2018	Japan	Ret	N/A	Regular clip	129/269	0.8 (1) vs. 6.7 (18) *p* = 0.019	N/A	N/A
Miyakawa et al. [51]	2021	Japan	Ret	25.7 ± 9.6/ 31.2 ± 16.4	Regular clip	275/339	2.2 (6) vs. 5.9 (20) *p* = 0.022	N/A	2.9 (8) vs. 0.9 (3) *p* = 0.115
Omori et al. [52]	2022	Japan	Ret	40.1 ± 12.1/ 47.7 ± 16.1	Regular clip	97/122	1.0 (1) vs. 6.6 (8) *p* = 0.088	0.0 (0) vs. 1.6 (2) *p* = 0.581	N/A
Takada et al. [53]	2025	Japan	Ret	DOAC 25 (20–32)/ 25 (20–30) ## Warfarin 25 (20–32)/ 25 (20–33) ##	Various clipping	212/212 82/82	5.2 (11) vs. 10.8 (23) *p* = 0.048 6.1 (5) vs. 17.1 (14) *p* = 0.049	N/A	N/A
Nishino et al. [54]	2025	Japan	Ret	35.4 ± 12.5/ 37.0 ± 13.6	Double-layered suturing	136/136	0.7 (1) vs. 4.4 (6) *p* = 0.120	0.0 vs. 0.0 *p* = 1.0	2.2 (3) vs. 5.1 (7) *p* = 0.335
Cristofaro et al. [55]	2026	Italy	Ret	1199/1943	Various clipping	1199/1943	7.2 (86) vs. 6.7 (130) *p* = 0.66	N/A	N/A
Maruo et al. [41]	2026	Japan	Ret	35.5 ± 9.0/ 37.5 ± 9.2	Traction device	55/136	1.8 (1) vs. 1.5 (2) *p* = 1.000	0.0 (0) vs. 2.2 (3) *p* = 0.558	3.6 (2) vs. 14.0 (19) *p* = 0.042

ESD: endoscopic submucosal dissection, SD: standard deviation, N/A: not applicable, DB: delayed bleeding, DP: delayed perforation, PECS: post-ESD coagulation syndrome, RCT: randomized control trial, Ret: retrospective study, * lesion size, ** lesion size, median (interquartile range: IQR), # lesion size, mean (range), ## lesion size, median (IQR), closure vs. partial-+non-closure.

**Table 4:** Efficacy of complete closure for DB, DP, and PECS according to 14 reports, including RCTs and retrospective studies.

	Closure	Non-Closure	*p* Value
Overall (14 studies)			
DB	4.3 (115/2694)	6.6 (247/3740)	<0.001
DP	0.6 (4/702)	1.6 (13/834)	0.109
PECS	7.5 (63/840)	8.5 (84/985)	0.421
RCT (4 studies)			
DB	0.9 (3/336)	4.9 (17/348)	0.004
DP	1.2 (4/336)	2.0 (7/348)	0.582
PECS	15.5 (50/323)	15.2 (49/323)	0.913
Ret (10 studies)			
DB	4.7 (112/2358)	6.8 (230/3392)	<0.001
DP	0.0 (0/366)	1.2 (6/486)	0.085
PECS	2.5 (13/517)	5.3 (35/662)	0.024

ESD: endoscopic submucosal dissection, DB: delayed bleeding, DP: delayed perforation, PECS: post-ESD coagulation syndrome, RCT: randomized control trial, Ret: retrospective study.

## Data Availability

The patient data used to support the findings of this study are available from the corresponding author upon request. However, some data is restricted by the institutional review board of the Kyoto Prefectural University of Medicine.

## Supplementary Materials

The following supporting information can be downloaded at: https://www.mdpi.com/article/10.3390/diagnostics16142148/s1, Table S1: The methodological quality of the 4 RCTs using the Cochrane Risk of Bias 2 tool, Table S2: The methodological quality of 18 retrospective studies according to the Newcastle-Ottawa scale.
