# Supplementary material for: Endoscopic Closure After Colorectal ESD: A Literature Review and Meta-Analysis on Its Efficacy in Preventing Adverse Events
Source: Diagnostics (Basel). 2026 Jul 8;16(14):2148. doi: 10.3390/diagnostics16142148 (PMC13408305; doi:10.3390/diagnostics16142148)
Supplement: Supplementary file 1 [file diagnostics-16-02148-s001.zip › diagnostics-4330911-supplementary.pdf]

Supplemental Table 1. The methodologic quality of the 4 RCTs according to the Cochrane Risk of Bias 2 tool.

Study	Design	Closure method	Cases Closure/Control	Randomization process	Deviations from intended interventions	Missing outcome data	Measurement of the outcome	Selection of the reported result	Overall risk of bias	Quality	Comments
Osada, et al. [44]	RCT	Regular clip	13/13	Some concerns	Low risk	Low risk	Low risk	Low risk	Some concerns	High	Small RCT with limited sample size.
Lee, et al. [45]	RCT	Regular clip	110/110	Low risk	Low risk	Low risk	Low risk	Low risk	Low risk	High	RCT; open-label design inherent to endoscopic closure.
Nomura, et al. [46]	RCT	Regular clip	71/84	Low risk	Low risk	Low risk	Low risk	Low risk	Low risk	High	RCT; open-label design inherent to endoscopic closure.
Miyakawa, et al. [47]	RCT	Regular clip	142/141	Low risk	Low risk	Low risk	Low risk	Low risk	Low risk	High	RCT; open-label design inherent to endoscopic closure.

RCT: randomized controlled trials

Supplemental Table 2. The methodologic quality of 18 retrospective studies according to the Newcastle-Ottawa scale

Study	Design	Closure method	Cases Closure/Control	Selection (max 4)	Comparability (max 2)	Outcome (max 3)	Total NOS score (0-9)	Quality	Comments
Fujiwara, et al. [48]	Ret	Regular clip	27/41	2	1	2	5	Moderate	Small exploratory retrospective cohort.
Yamasaki, et al. [25]	Ret	Line and clip	51/51	2	1	2	5	Moderate	Small exploratory retrospective cohort.
Ogiyama, et al. [49]	Ret	Regular clip	95/61	3	1	2	6	Moderate	Retrospective observational cohort.
Yamamoto, et al. [50]	Ret	Regular clip	129/269	3	1	2	6	Moderate	Retrospective observational cohort.
Miyakawa, et al. [51]	Ret	Regular clip	275/339	3	1	2	6	Moderate	Retrospective observational cohort.
Omori, et al. [52]	Ret	Regular clip	97/122	3	1	2	6	Moderate	Retrospective observational cohort.
Takada, et al. [53]	Ret	Various clipping	212/212	3	2	2	7	High	Moderate-sized retrospective cohort with improved comparability.
Nishino, et al. [54]	Ret	Double-layered suturing	136/136	3	2	2	7	High	Moderate-sized retrospective cohort with improved comparability.
Cristofaro, et al. [55]	Ret	Various clipping	1199/1943	4	2	2	8	High	Large multicenter retrospective cohort with comparative methodology.
Maruo, et al. [41]	Ret	Traction device	55/136	2	1	2	5	Moderate	Small exploratory retrospective cohort.

Ret: retrospective studies
